# An Emerald-Colored Intoxication: A Case of Sulfhemoglobinemia Induced by Metoclopramide

**DOI:** 10.7759/cureus.90639

**Published:** 2025-08-21

**Authors:** Sarah Degrève, Céline Rousseaux, Carnot Tanie Talom

**Affiliations:** 1 Emergency Department, CHIREC (Centre Hospitalier Interrégional Edith Cavell) – Braine l’Alleud Hospital, Braine l’Alleud, BEL; 2 Emergency Department, CHIREC (Centre Hospitalier Interrégional Edith Cavell) – Braine l’Alleud Hospital, Braine l'Alleud, BEL

**Keywords:** cyanosis, dyshemoglobinemia, intoxication, metoclopramide, sulfhemoglobin

## Abstract

Sulfhemoglobinemia (SulfHb) is a rare condition characterized by the presence of sulfhemoglobin in the blood, resulting from the incorporation of a sulfur atom into the hemoglobin molecule. This alteration prevents hemoglobin from effectively binding and transporting oxygen, leading to clinical symptoms such as cyanosis and tissue hypoxia.

We report the case of a 59-year-old female patient who was admitted to the emergency department for desaturation. The patient presented with persistent lip cyanosis and exertional dyspnea over several months. Blood analyses revealed leukocytosis and the detection of sulfhemoglobin. A diagnosis of SulfHb induced by metoclopramide intoxication was established. The treatment involved discontinuing metoclopramide and administering oxygen. The patient was subsequently monitored monthly during outpatient consultations, and the sulfhemoglobin levels were no longer detectable three months after discharge.

This case highlights the synergy between metoclopramide overdose, CYP2D6 inhibition by duloxetine, and exposure to sulfur donors in promoting sulfhemoglobin formation. It underscores the importance of a systematic medication review and the early use of co-oximetry in any unexplained desaturation to avoid diagnostic and management delays. The case underscores the importance of vigilance in prescribing and monitoring pharmacological treatments. Rapid recognition of SulfHb and the utilization of appropriate diagnostic tools are essential to ensure optimal patient care.

## Introduction

Sulfhemoglobinemia (SulfHb) is a rare but potentially serious condition marked by the presence of sulfhemoglobin in the blood. Sulfhemoglobin is a modified form of hemoglobin resulting from the incorporation of a sulfur atom into its structure. This modification reduces hemoglobin’s capacity to bind and transport oxygen, leading to clinical manifestations such as cyanosis and tissue hypoxia. SulfHb can arise from exposure to specific oxidizing drugs, such as metoclopramide, as well as to sulfur-containing agents.

Diagnosis typically relies on co-oximetry and may require a cyanide differentiation test to distinguish sulfhemoglobin from methemoglobin. Treatment is supportive.

In this case report, we describe a 59-year-old patient who developed SulfHb after prolonged metoclopramide use. This case highlights the diagnostic and therapeutic challenges of this rare condition and underscores the need for vigilant prescribing and treatment monitoring.

## Case presentation

A 59-year-old woman was referred to the emergency department by her primary care physician for acute desaturation. Three weeks earlier, during an anesthesiology consultation in preparation for an epidural, her oxygen saturation had been noted at 80%. Her general practitioner (GP) subsequently prescribed Medrol and home oxygen therapy. One week later, she saw a pulmonologist, who arranged spirometry for the following month; at that time, her saturation had improved to 93%. However, one week after that appointment, the patient returned to her GP with a room air saturation of 74%, which led to her admission to the emergency department.

The patient had experienced exertional dyspnea for several months, with no other significant complaints on history taking. Her husband, however, reported persistent lip cyanosis over the same period.

Her medical history included depression, hypothyroidism, hypercholesterolemia, untreated chronic obstructive bronchitis, and abdominal obesity, as well as fibromyalgia, complex regional pain syndrome, de Quervain’s tenosynovitis, and hip pain. She had previously undergone an appendectomy, inguinal hernia repair, and lumbar arthrodesis.

The patient was on multiple chronic medications and supplements, including duloxetine 120 mg once daily, rosuvastatin 20 mg once daily, pantoprazole 40 mg once daily, Cystiphane, metoclopramide 10 mg as needed (up to eight tablets per 24 h), Medrol (recently started), paracetamol, acetylsalicylic acid 80 mg, levothyroxine 75 µg, extended-release alprazolam 1 mg, amitriptyline 25 mg three times daily, diazepam 10 mg, etilefrine, and various vitamins and minerals.

Upon arrival in the emergency department, her vital signs were: SpO₂ 84% on room air, blood pressure 204/121 mmHg, heart rate 103 bpm, temperature 36.4 °C, and respiratory rate 20 breaths/min. Physical examination revealed facial, lip, and extremity cyanosis, along with digital clubbing. She also displayed Cushingoid features, including lower-limb muscle atrophy, telangiectasias, ecchymoses on the legs, and a buffalo hump. Cardiac, pulmonary, and abdominal auscultation were unremarkable.

Laboratory results (Table 1) revealed a marked leukocytosis of 17,800/µL with 16,800 neutrophils. Hemoglobin was 13.3 g/dL, with a normal mean corpuscular volume (MCV) and slightly elevated haptoglobin. Inflammatory markers, renal function, and liver enzymes were within normal limits. Cardiac troponins were negative.

**Table 1:** Laboratory results g/dL: gram per deciliter; fL: femtoliters; g/L: gram per liter; cells/µL: cells per microliter; mg/dL: miligram per deciliter; U/L: unit per liter; ng/L: nanogram per liter

Parameter	Abbreviation	Results	Reference Range
Hemoglobin	Hb	13.3	11.7 – 17.4 g/dL
Mean Corpuscular Volume	MCV	100	80 – 100 fL
White blood cells	WBC	17.8	4.5 – 11.0 1000 cells/µL
Neutrophils	Neutrophils	16.8	1.8 – 7.7 1000 cells/µL
Haptoglobin	Haptoglobin	2.3	0.3 – 2.0 g/L
C-reactive protein	CRP	5	< 5 mg/dL
Glutamic-Oxaloacetic Transaminase	GOT	30	< 40 U/L
Glutamate-Pyruvate Transaminase	GPT	25	< 41 U/L
Creatinine	Creatinine	0.95	0.70 – 1.20 mg/dL
Urea	Urea	23	18 – 55 mg/dL
High-Sensitivity Troponin T	Troponin T HS	8	< 14 ng/L

Arterial blood gas analysis (Table 2) showed a normal acid-base balance and oxygenation, with a mildly increased lactate level. An alert was triggered due to interference suggestive of SulfHb.

**Table 2:** Blood gas analysis mmHg: millimeters of mercury; mmol/L: millimoles per liter

Parameter	Abbreviation	Results	Reference Range
pH	pH	7.45	7.35 – 7.45
Partial pressure of CO₂	pCO₂	36	35 – 48 mmHg
Partial pressure of O₂	pO₂	84	83 – 108 mmHg
Lactate	Lactate	2.1	0.36 – 0.75 mmoL/L
Bicarbonate	HCO3-	22	21.0 – 28.0 mmol/L

Based on her medication history, SulfHb due to metoclopramide toxicity was promptly diagnosed. She was transferred to the pulmonology ward, where she remained for 11 days. Management consisted of stopping metoclopramide and administering oxygen at 60% fraction of inspired oxygen (FiO_2_) initially, which was tapered to 1 L/min (≈24%) at discharge. Monthly outpatient follow-up revealed that sulfhemoglobin was no longer detectable three months after discharge, although exact levels could not be determined.

## Discussion

Adult human hemoglobin (HbA) comprises four heme groups, each made up of a porphyrin ring and an iron atom. The porphyrin itself consists of four pyrrole rings. Sulfhemoglobin is a green‐pigmented molecule that forms when ferrous iron (Fe²⁺) is irreversibly oxidized to ferric iron (Fe³⁺) through the incorporation of a sulfur atom into one of the pyrrole rings, permanently altering the heme’s three‐dimensional structure [1-3]. This modification prevents the molecule from binding oxygen and cannot be chemically reversed; sulfhemoglobin remains present until the red blood cell containing it is naturally cleared, about 120 days after its formation [2,3].

It was long believed that sulfhemoglobin did not occur physiologically in the body. However, it is now clear that the human body naturally produces hydrogen sulfide (H₂S), an important physiological mediator involved, for example, in modulating neuronal activity and promoting muscle relaxation. One regulatory pathway for this endogenous H₂S involves its binding to certain heme proteins, forming a moderate, non‐pathological sulfheme complex. This mechanism is thought to represent one route of H₂S catabolism, albeit at the cost of irreversible heme alteration and progressive elimination through erythrocyte clearance [2].

High levels of sulfhemoglobin can be potentially toxic because the molecule cannot bind oxygen, which may lead to SulfHb--a rare cause of cyanosis induced by certain oxidizing drugs (sulfur-containing or not) and by exposure to sulfurous products [1,2]. In our case report, chronic excessive use of metoclopramide was identified as the primary trigger for SulfHb. This drug has also been implicated in other case reports [4,5].

Metoclopramide is primarily metabolized via the CYP2D6 enzymatic pathway [6]. In this patient, concomitant administration of duloxetine, a well-documented moderate inhibitor of that cytochrome, likely led to partial inhibition of this route [7]. This pharmacokinetic interaction could have caused a marked accumulation of metoclopramide in plasma. Although this concentration increase could not be confirmed by assay, it may have created an environment conducive to unusual reactions, such as heme oxidation.

The exact origin of the sulfur atom incorporated into sulfhemoglobin remains unclear, but several drug-related sulfur sources can be considered in this case. In addition to H₂S potentially released by the intestinal flora or glutathione [1,5,8,9], the patient was taking compounds such as cystine (metabolized to cysteine, rich in sulfhydryl groups), pantoprazole (containing a sulfinyl moiety), and duloxetine (whose thiophene ring also represents a possible sulfur source). This combined exposure may have created a biochemical environment sufficient for sulfhemoglobin formation in the presence of oxidized heme.

This pathophysiological hypothesis is supported by other cases reported in the literature, notably in connection with N-acetylcysteine [4,5].

From a clinical standpoint, the functional alteration of hemoglobin in SulfHb presents as a form of pseudo-anemia, since a portion of the hemoglobin is rendered unable to bind oxygen. The resulting cyanosis isn’t caused by an increase in deoxyhemoglobin (HHb), as seen in cardiac or pulmonary disease, but by an irreversible change in hemoglobin’s pigmentation [1,3,9].

The hemoglobin dissociation curve, which illustrates the relationship between oxygen partial pressure and hemoglobin saturation, is a valuable tool for understanding the functional impact of sulfhemoglobin. Although SulfHb markedly reduces overall oxygen transport, it also lowers the affinity of the remaining healthy heme sites by diminishing the molecule’s allosteric cooperativity. This shifts the dissociation curve to the right, thereby enhancing oxygen release into peripheral tissues [1-3].

In comparison, methemoglobinemia or carbon monoxide (CO) poisoning produces an opposite leftward shift of the curve, increasing hemoglobin’s residual affinity for oxygen and impairing tissue delivery. Thus, although SulfHb is clinically marked by striking cyanosis, it generally leads to less severe tissue hypoxia than these other hemoglobin alterations.

Comparison of different hemoglobin alterations

Hemoglobin alterations can arise from various causes and carry different clinical implications. Below is a comparison of the main forms of hemoglobin alteration.

Methemoglobinemia (MetHb)

MetHb forms by oxidation of Fe²⁺ to Fe³⁺, rendering hemoglobin incapable of binding oxygen. It results from exposure to oxidizing agents, including certain drugs sometimes also implicated in SulfHb. As with SulfHb, MetHb causes cyanosis and tissue hypoxia. It also alters the behavior of residual healthy Hb by increasing its affinity for O₂ and shifting the dissociation curve to the left, thereby leading to tissue hypoxia [1,3,9].

Spectrophotometrically, MetHb absorbs more infrared light than oxyhemoglobin (O₂Hb) or deoxyhemoglobin (HHb), but absorbs red light similarly to HHb, explaining the clinical cyanosis and the characteristic dark (“chocolate”) appearance of the blood [1].

Treatment involves removing the causative agent, providing supportive care, and administering a reducing agent (methylene blue), bearing in mind that the dye in this molecule can distort oxygen saturation (SpO₂) readings [1,3].

Carboxyhemoglobinemia (COHb)

Carbon monoxide (CO) is a colorless, odorless, non‐irritating gas produced by the incomplete combustion of organic materials (wood, coal, natural gas, etc.) [1]. It binds to hemoglobin with an affinity approximately 240 times greater than that of oxygen, forming carboxyhemoglobin (COHb) [1]. This binding not only prevents O₂ from attaching at the occupied site but also alters hemoglobin’s allosteric structure, reducing the ability of the remaining sites to release oxygen to peripheral tissues. As a result, the oxygen-hemoglobin dissociation curve shifts to the left.

Clinical manifestations are varied and often nonspecific: headache, dizziness, confusion, vomiting, etc. Treatment primarily involves administering high‐concentration oxygen (normobaric or hyperbaric, depending on severity) to accelerate CO dissociation from hemoglobin and restore adequate tissue oxygenation [10].

Sulfhemoglobinemia (SulfHb)

As described previously, SulfHb results from the irreversible oxidation of Fe²⁺ to Fe³⁺ through the incorporation of a sulfur atom. Unlike the other two hemoglobin alterations, SulfHb shifts the hemoglobin dissociation curve to the right, reflecting decreased O₂ affinity and enhanced oxygen release in peripheral tissues, which explains its often better clinical tolerance despite marked cyanosis. Because this oxidation is irreversible, no specific antidote exists. Management involves discontinuing the causative agent and providing symptomatic care. SulfHb levels then decline gradually with red blood cell turnover, approximately 120 days after their formation [1,2,8].

These various hemoglobin alterations are summarized in Table 3 [1,2,3,8-10].

**Table 3:** Comparison of hemoglobin alterations

Type of alteration	Mechanism	Effect on the oxygen–hemoglobin dissociation curve	Clinical features	Treatment
Methemoglobinemia (MetHb)	Reversible oxidation of Fe²⁺ to Fe³⁺	Leftward shift of the dissociation curve	Cyanosis, tissue hypoxia, chocolate-brown blood	Methylene blue, removal of the causative agent, and supportive care
Carboxyhemoglobinemia	Binding of CO to hemoglobin (240× affinity vs O₂)	Leftward shift of the dissociation curve	Headache, dizziness, confusion, nausea, tachycardia, hypotension, cherry-red skin and blood	Normobaric or hyperbaric oxygen therapy
Sulfhemoglobinemia	Irreversible incorporation of a sulfur atom into oxidized heme (Fe³⁺)	Rightward shift of the dissociation curve	Cyanosis with greenish-colored blood, usually only mild dyspnea or fatigue	Discontinuation of the causative agent, symptomatic treatment, and gradual elimination via red blood cell turnover

Methods of detection and measurement

To evaluate oxygen saturation and detect hemoglobin anomalies, two primary tools are employed: the pulse oximeter and the co-oximeter.

The pulse oximeter operates on the principle that oxyhemoglobin (O₂Hb) and deoxyhemoglobin (HHb) absorb red light (660 nm) and infrared light (940 nm) to differing degrees. It relies on a simplified mathematical model that presumes only these two hemoglobin species are present. When altered hemoglobins (MetHb, SulfHb, COHb) are present, each absorbing light in an atypical manner, SpO₂ readings become unreliable, often plateauing around 85% regardless of the true saturation level [3].

Co-oximeters, by contrast, perform direct spectrophotometric analysis on a blood sample, typically measuring four hemoglobin species: O₂Hb, HHb, COHb, and MetHb. However, a fifth species, such as sulfhemoglobin, is not specifically detected by these instruments [4]. SulfHb is often mistaken for MetHb because their absorption bands lie close together--618 nm for SulfHb [2] versus 630 nm for MetHb [4,11]. This spectral proximity can lead to a “pseudomethemoglobinemia.” To distinguish them, a cyanide test may be employed: adding cyanide forms a complex with MetHb (cyanomethemoglobin), causing its absorption band to disappear, while the SulfHb band remains unchanged [11].

Ultimately, this case lays bare how the convergence of a metoclopramide overdose, duloxetine-mediated CYP2D6 inhibition, and simultaneous exposure to sulfur-donor compounds created the perfect milieu for sulfhemoglobin formation. It highlights the necessity of a thorough review of all medications and the early use of co-oximetry in any episode of unexplained desaturation, particularly in patients on multiple drugs.

## Conclusions

This case of metoclopramide-induced sulfhemoglobinemia (SulfHb) highlights the dangers of polypharmacy and pharmacokinetic interactions, which can lead to rare but potentially serious alterations of hemoglobin. Although uncommon, SulfHb presents with cyanosis that is refractory to conventional approaches and demands prompt recognition to avert severe complications. The use of appropriate diagnostic tools--most notably co-oximetry--is crucial for swiftly distinguishing among dyshemoglobinemias (MetHb, COHb, SulfHb) and guiding management. To prevent such adverse events, it is essential to conduct a comprehensive medication review, anticipate cytochrome-mediated interactions, strengthen ongoing prescriber education, and maintain active pharmacovigilance. Only a multidisciplinary approach, combining clinical rigor, interdisciplinary collaboration, and close monitoring of patients on complex regimens, can ensure their safety and secure optimal care.
